# Predictive modeling of plant messenger RNA polyadenylation sites

**DOI:** 10.1186/1471-2105-8-43

**Published:** 2007-02-07

**Authors:** Guoli Ji, Jianti Zheng, Yingjia Shen, Xiaohui Wu, Ronghan Jiang, Yun Lin, Johnny C Loke, Kimberly M Davis, Greg J Reese, Qingshun Quinn Li

**Affiliations:** 1 Department of Automation, Xiamen University, Xiamen, Fujian, 361005, P. R. China; 2 Department of Botany, Miami University, Oxford, OH 45056, USA; 3 Research Computing Group, IT Services, Miami University, Oxford, OH 45056, USA; 4 Current address: Department of Medicine, Division of Liver Diseases, Mount Sinai Medical Center, 1425 Madison Avenue, RM 1176, New York, NY 10029, USA

## Abstract

**Background:**

One of the essential processing events during pre-mRNA maturation is the post-transcriptional addition of a polyadenine [poly(A)] tail. The 3'-end poly(A) track protects mRNA from unregulated degradation, and indicates the integrity of mRNA through recognition by mRNA export and translation machinery. The position of a poly(A) site is predetermined by signals in the pre-mRNA sequence that are recognized by a complex of polyadenylation factors. These signals are generally tri-part sequence patterns around the cleavage site that serves as the future poly(A) site. In plants, there is little sequence conservation among these signal elements, which makes it difficult to develop an accurate algorithm to predict the poly(A) site of a given gene. We attempted to solve this problem.

**Results:**

Based on our current working model and the profile of nucleotide sequence distribution of the poly(A) signals and around poly(A) sites in Arabidopsis, we have devised a Generalized Hidden Markov Model based algorithm to predict potential poly(A) sites. The high specificity and sensitivity of the algorithm were demonstrated by testing several datasets, and at the best combinations, both reach 97%. The accuracy of the program, called *p*oly(*A*) *s*ite *s*leuth or *PASS*, has been demonstrated by the prediction of many validated poly(A) sites. *PASS *also predicted the changes of poly(A) site efficiency in poly(A) signal mutants that were constructed and characterized by traditional genetic experiments. The efficacy of *PASS *was demonstrated by predicting poly(A) sites within long genomic sequences.

**Conclusion:**

Based on the features of plant poly(A) signals, a computational model was built to effectively predict the poly(A) sites in Arabidopsis genes. The algorithm will be useful in gene annotation because a poly(A) site signifies the end of the transcript. This algorithm can also be used to predict alternative poly(A) sites in known genes, and will be useful in the design of transgenes for crop genetic engineering by predicting and eliminating undesirable poly(A) sites.

## Background

Eukaryotic messenger RNA (mRNA), after being transcribed from its coding gene, typically undergoes processing events, such as capping, splicing, and polyadenylation, before it is translocated to the cytoplasm and translated into proteins. While these three essential steps of processing are interrelated, each step is performed by a defined set of protein factors and uses specific signals encoded in the precursor mRNA (pre-mRNA) [1]. The polyadenylation signals for all eukaryotes seem to have three common parts: a cleavage site (CS), a near upstream element (called NUE in plants, equivalent to AAUAAA in animals) about 20–30 nucleotides (nt) upstream of the CS, and an element about 50 nt upstream of the CS (termed far upstream element or FUE in plants) [2-4]. In mammals, there is an additional signal element located ~20 nt downstream of the CS [3], which is not commonly observed in yeast and plants. Moreover, both yeast and plants possess much less sequence conservation in NUE and FUE regions compared to that of animals [4-6]. However, there is little conservation between yeast and plants in term of sequences of the poly(A) signal elements.

Plant polyadenylation signals in general are more similar to yeast, in which no highly conserved signal sequences have been identified. For example, a recent work revealed that the NUE signal AAUAAA, albeit proven the best signal in plants [7], can only be found at the right position in about 10% of Arabidopsis genes [6]. However, the same signal is used by over 50% of human genes [4]. This makes it very difficult to predict the CS of plant genes without experimental evidence such as EST that can be used to deduce poly(A) sites. With many ongoing plant genome sequencing projects, using poly(A) sites as a determinant of the 3'-end of genes would greatly enhance the accuracy of genome annotation. To this end, we were interested in devising an algorithm to predict poly(A) sites using our newly developed nucleotide composition model of poly(A) signals in 3'-UTR of the genes in the model plant Arabidopsis [6].

In our improved plant polyadenylation signal model, there are three types of sequence elements that possess some level of conservation, FUE, NUE, and the newly defined cleavage element (CE) [6]. Within the CE, there are three sub-domains made up of different prevailing sequences: the highly conserved di-nucleotide (CA and UA) right before the CS; and two U-rich sequence elements on the right and the left sides of CS, termed CE-R and CE-L. Beyond signal sequence information, the clear transition of the nucleotide composition [6] also offers additional features for the design of the algorithm. Briefly, a high U/A ratio in the FUE decrease to a into a low value (high A/U ratio) in the NUE. Such a transition happens two more times between the NUE and CE, and within the CE. Finally, the U/A ratio becomes 1 beyond 50 nt downstream of the CS. During these U/A transitions, the G and C contents remain low except at the CS where a spike of C is evident [6]. Such a profile of the 3'-UTR in Arabidopsis has been confirmed independently [8]. Other features of Arabidopsis polyadenylation signals are also found in that of the rice genome [9].

The Hidden Markov Model (HMM), a widely used system in bioinformatics, is a probability-based mathematical model with a complete set of theory, methods and an algorithm. It is widely used to describe both stability and variability of signals over background. Rabiner [10] systematically described the HMM and made it a common technology in voice recognition. In recent years, because of the similarity of biological data (DNA, RNA and protein sequences) to voice signals, the HMM has been used in different aspects of sequence analysis such as sequence comparison, prediction of protein structures and gene annotation. However, the length of the state in the original HMM is geometrically distributed, which limits its application. A new generation of the HMM, called the Generalized Hidden Markov Model (GHMM; [10-12]), was introduced to extend the utility of the HMM. The GHMM gives each state multiple observed values (instead of the single value in the HMM), so it can easily be used in describing the organization of gene sequences. In this paper, we present a GHMM-based method for predicting the poly(A) sites in Arabidopsis. The prediction results of poly(A) sites are compared with experimentally validated data for some of the genes. Interestingly, our program can also predict the results of traditional mutation studies, as the site efficiencies and scores given by the program are linearly correlated.

## Results

We were interested in using a computer program to predict the plant poly(A) site in a given transcript (for convenience, presented as a DNA sequence). To do so, we transformed the profiles of the known poly(A) sites and their adjacent region, based on the data presented by Loke et al. [6] from Arabidopsis, into features that can be used for computational modeling. The analysis of a dataset of 8160 sequences (hereafter called 8K dataset) described in that paper provided the basis for setting parameters as described in the Methods section. Hence, we designed an algorithm and wrote a code named *Poly(A) Site Sleuth *(or *PASS*) in PASCAL.

### Sensitivity and specificity of the program

To evaluate the performance of *PASS*, we employed the two most common standards: sensitivity (*Sn*) and specificity (*Sp*). The definitions are:

Sn=TPTP+FNSp=TPTP+FP=1−FPTP+FP
 MathType@MTEF@5@5@+=feaafiart1ev1aaatCvAUfKttLearuWrP9MDH5MBPbIqV92AaeXatLxBI9gBaebbnrfifHhDYfgasaacH8akY=wiFfYdH8Gipec8Eeeu0xXdbba9frFj0=OqFfea0dXdd9vqai=hGuQ8kuc9pgc9s8qqaq=dirpe0xb9q8qiLsFr0=vr0=vr0dc8meaabaqaciaacaGaaeqabaqabeGadaaakeaafaqabeqacaaabaGaem4uamLaemOBa4Maeyypa0ZaaSaaaeaacqWGubavcqWGqbauaeaacqWGubavcqWGqbaucqGHRaWkcqWGgbGrcqWGobGtaaaabaGaem4uamLaemiCaaNaeyypa0ZaaSaaaeaacqWGubavcqWGqbauaeaacqWGubavcqWGqbaucqGHRaWkcqWGgbGrcqWGqbauaaGaeyypa0JaeGymaeJaeyOeI0YaaSaaaeaacqWGgbGrcqWGqbauaeaacqWGubavcqWGqbaucqGHRaWkcqWGgbGrcqWGqbauaaaaaaaa@4E60@

In these equations, *TP *(true positive) is the number of actual sites that are identified or predicted correctly. *FN *(false negative) is the number of actual sites that cannot be identified or predicted correctly. *FP *(false positive) is the number of false sites that are predicted by *PASS*. The value of *Sn *represents the fraction of the actual poly(A) sites that can be predicted, while *Sp *represents the fraction of actual poly(A) sites in all the predicted sites. The higher the *Sp *value is, the lower the fraction of false positive sites among predicted sites is.

**Figure 1:**
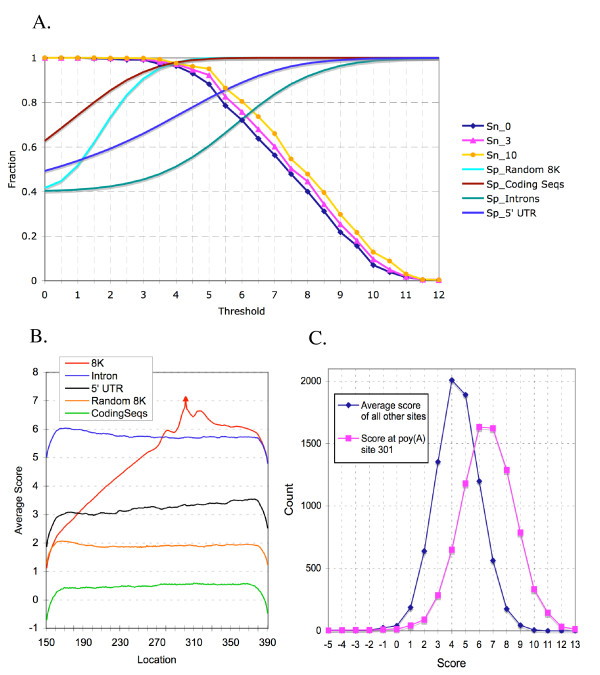
Assessment of the algorithm and *PASS *program. A. The relationship of sensitivity (*Sn*), specificity (*Sp*), and threshold. Threshold is a selectable standard in determining whether a poly(A) site is next to a nucleotide or not. It is also measured as a score for each nucleotide of an individual sequence. The higher the threshold, the better the probability that a nucleotide is a poly(A) site. *Sn*_0, *Sn*_3, and *Sn*_10 represent the distance between the prediction site and the validated site to be 0, 3, and 10 nucleotides, respectively. Random 8K, a randomly generated 8000 sequence dataset based on the 2^nd ^order distribution of trinucleotide in the 8K dataset. Coding Seqs, 8000 coding sequences from Arabidopsis (downloaded from TAIR). Intron (8000 sequences) and 5'-UTR (974 sequences) datasets are also from Arabidopsis. B. The average prediction scores of the 8K dataset and other control datasets as in A. The authenticated poly(A) site at location 301 is as mark by a red triangle. C. Distribution of scores in the 8K dataset. The distribution of all other sites (except position 301) is presented as average scores of all these sites. The scores at the 301 position of each of the sequences were counted and their distribution of them is presented.

To evaluate the algorithm, we tested 568 known poly(A) sites (randomly chosen from the 8K dataset described in [6] and the Methods) to calculate *Sn*. Because not all poly(A) sites have been identified in each sequence of the database, we cannot calculate the real *Sp *value. Therefore, we used several negative control datasets for *Sp *calculations. These include Arabidopsis 5' UTRs, introns, coding sequences, and a randomly generated sequence dataset that preserve the trinucleotide distributions found in the 8K dataset. Since all the sites predicted by *PASS *in these control sequences are false sites, *FP *was set to be the number of sites that were predicted in these sequences. (*TP*+*FP*) was set to be the total number of sites. The results are shown in Figure 1A. The horizontal value represents the threshold, which is an user selectable standard in determining whether or not a nucleotide is a poly(A) site. If the value of a nucleotide is higher than the threshold, this position is thought to be a poly(A) site. *Sn*_0, *Sn*_3, and *Sn*_10 represent the distance between the predicted site and the known site, which are 0, 3 and 10 nucleotides, respectively. *Sn*_0 means that the predicted site is exactly the same as the known poly(A) site (0 distance). Based on Figure 1A, when the threshold is increased, *Sn *decreases while *Sp *increases. There is no drastic different when the *Sn *are calculated with the three positions relative to the poly(A) sites. However, *Sp *can be quite different when different control sequences are used. For the coding sequences and the randomly generated sequences, both *Sn *and *Sp *reach 97% at a threshold 4. For 5' UTR, *Sn *and *Sp *are about 82% at a threshold of 5.2. In the intron sequences, *Sn *and *Sp *are lower than others at 72% at a threshold of 6. The lower *Sp *may reflect the feature of the sequences of 5'UTR and the introns, because these sequences tend to have higher A and T content, a characteristic shared by 3' UTR on which *PASS *design was based.

To evaluate the program, we tested the sequences in the above-mentioned datasets. Using the probability score, an output of *PASS*, we examined the distributions of the scores as shown in Figure 1B. The average scores of the 8K dataset peak at location 301, the authenticated poly(A) site position in these sequences. This is a demonstration of the efficacy of the program because it was designed to predict these positions as poly(A) sites. The average scores of the control sequences are much lower than that of 8K with the exception of the intron dataset. Again, this could be due the shared features between 3' UTR and the introns. Importantly, there is 1 point score difference between the average peak score of the poly(A) sites and that of the introns, which is significant enough to differentiate the poly(A) site from introns. This is demonstrated in the genomic sequence scan that is discussed later.

To further examine the prediction scores that are distinctive for poly(A) sites, the distributions of the scores at position 301 and the average scores of all other non-poly(A) sites in all the sequences of 8K dataset were compared (Figure 1C). The majority of the poly(A) sites have a score between 6 and 7, whereas the average scores of all other non-poly(A) sites peak at 4–5. Difference of 1 to 2 score points again could be significant enough to resolve the poly(A) site from the background at the sequence level.

### Predicting poly(A) sites by PASS

**Figure 2:**
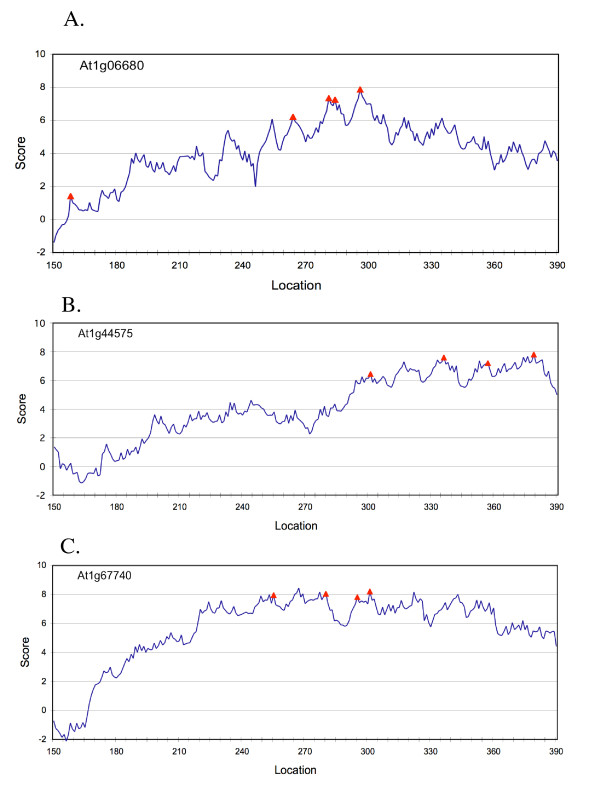
Representative outputs of the software using sequences with multiple poly(A) sites. The triangles indicate the poly(A) sites confirmed by EST data. The majority of the real sites have relatively high probability (scores). However, in some cases (e.g., the first site in A) there are low prediction value sites. See text for more detail. Locations of the horizontal axis indicate the relative positions of the poly(A) sites in the sequence.

To demonstrate the efficacy of the algorithm and the software, we tested many sequences including those with multiple poly(A) sites. Three of the typical results are shown graphically in Figure 2. In general, most of the experimentally authenticated poly(A) sites are found in the high probability area with scores larger than or around 6. However, not all predicted sites with high scores are confirmed by EST data. There are a few possible reasons for this. First, the EST data may not be exhaustive, meaning that not all sites have been found in the available EST dataset. Second, not all possible sites are efficiently used in the cells. Instead, some sites may only be used under certain environmental or developmental conditions. Third, some may be inaccurately predicted. This could be corrected by further optimization of the algorithm. It is very interesting to note that in several cases, there are authenticated poly(A) sites located in the low score area (e.g. the first site in Figure 2A, with a score around 2). The reason for this is not clear. One possible explanation could be that the use of this site could be facilitated by yet unknown trans-acting factors.

### Identification of multiple poly(A) sites

**Figure 3:**
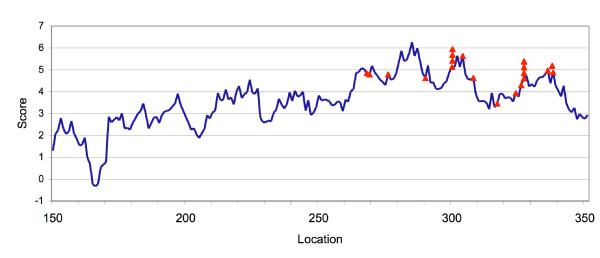
Comparison of *PASS *prediction and the validated poly(A) sites of a tobacco RNA binding protein-30 gene X65118. The triangles indicate the authenticated poly(A) sites, and the number of the triangles at one position denote the number of times cDNAs associated with the specific poly(A) site were found [13].

To see if *PASS *can differentiate multiple poly(A) sites, we further tested some genes that have been reported by others or collected from GenBank collections (e.g. NCBI's Unigenes). Tobacco RNA binding protein-30 gene (Accession# X65118), which is known as a gene with many alternative poly(A) sites [13], was scanned for poly(A) site scores by *PASS*. As shown in Figure 3, most of the poly(A) sites are in the highly scored (around 5) area of the 3'-UTR with a couple of exceptions (<4). However, the *PASS *predicted peaks at around location 280 were not validated. It is very likely that there are other factors contributing to the site selection, e.g. protein factors, RNA secondary structures, etc.

### Prediction of mutational alterations of poly(A) site efficiencies

**Figure 4:**
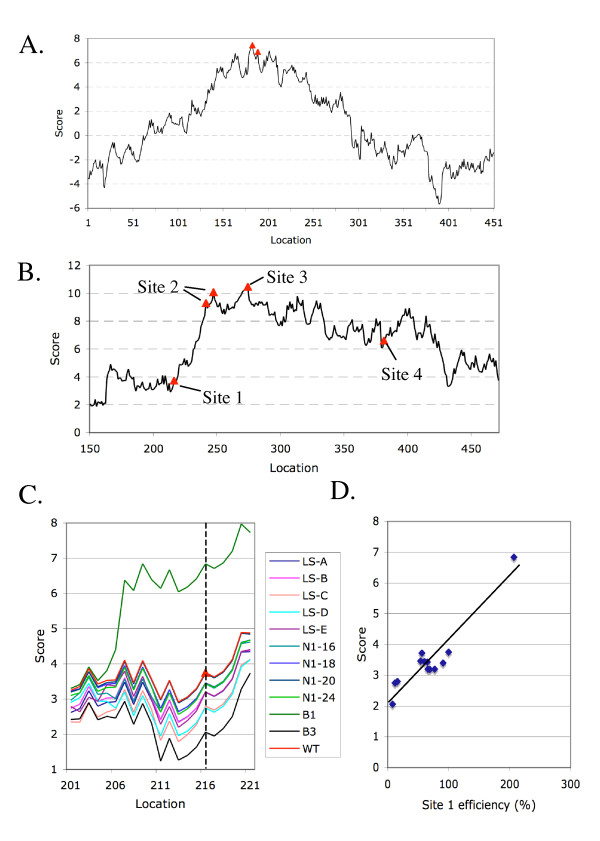
*PASS *predicted scores of well-studied poly(A) signals and the relative efficiencies of polyadenylation signal mutants of *rbcS *determined by wet experiments. The red triangles denote validated poly(A) sites in the wild type mRNA. A. *PASS *scan of CaMV 35S RNA 3'-UTR [14, 28], which is widely used as a polyadenylation signal for transgene expressions. B. Wild type 3'-UTR of *rbcS *profile scanned by *PASS*. The authenticated poly(A) sites are as marked. The predicted scores and the actual efficiencies of each site being used are tightly associated in which sites 2 and 3 are the major ones, while sites 1 and 4 are minor ones [7]. C. A set of representative poly(A) signal mutations of the site 1 of *rbcS *and their predicted scores by *PASS*. The dash line indicates the poly(A) site positions. D. Relationship between predicted score and poly(A) site 1 efficiencies of the mutants shown in C. The poly(A) site efficiency data were extracted from the results presented by Li and Hunt ([7]; Figures 2 to 4 therein).

One way to further assess the software would be to see if it can predict the change of the utility of poly(A) sites after the polyadenylation signals are mutated. Examples of this can be found in the well-studied 3'-UTRs. The polyadenylation signals for two genes, pea rubisco small subunit gene (*rbcS*) E-9 and cauliflower mosaic virus (CaMV) 35S transcript, have been extensively studied by classical mutagenesis and genetic means [2,6,7,14], and are being used widely in transgene constructions [15,16]. As shown on Figure 4A, the main poly(A) sites of the CaMV 3'UTR are located on the peak of the scores predicted by *PASS*. There are four validated poly(A) sites in *rbcS*, but sites 2 and 3 are the major poly(A) sites [17] (Figure 4B). Interestingly, our program predicted such site usage bias by 6–7 score points (compare to site 1). Again, similar to Fig. 3, there are a few peaks (meaning good poly(A) sites) after site 3 that are not used, presumably due to unknown factors that are not considered in this algorithm. It may also be possible that these sites are behind the major sites, and thus being skipped. Nonetheless, our predicted sites are typically in the near vicinity of the authenticated sites.

Detailed conventional mutagenesis experiments were performed on the poly(A) signal for *rbcS *site 1, which was chosen to avoid the overlapping signals of sites 2 and 3 [7]. Linker scanning, base substitution, and enhancing the signal by using AAUAAA all altered the site usage at different levels [7]. Interestingly, these changes can also be predicted by our software as indicated in Figure 4C. This becomes evident when the *PASS *scores are compared with the site efficiency (the fraction of a poly(A) site being chosen and used in the pool of the *rbcS *mRNA) after mutation (Figure 4D). There is a tendency of linear relationship between *PASS *scores and site efficiencies following mutation. This suggests that our model can identify the poly(A) sites both qualitatively and may be also quantitatively.

### Predicting poly(A) sites in the genomic sequences

**Figure 5:**
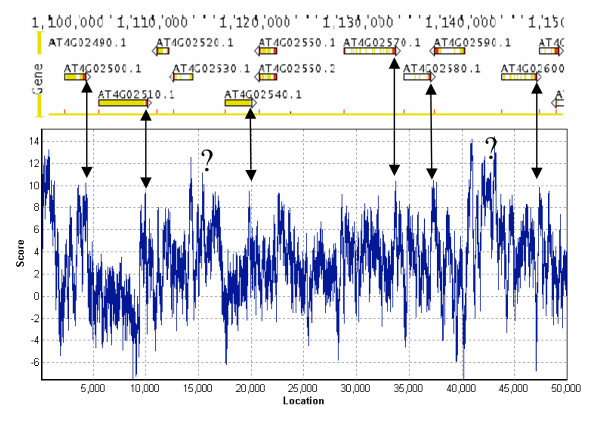
A representative result of using *PASS *to scan a segment of the genomic sequence of Arabidopsis. The top part of the image was downloaded (screen shot) from TAIR web page (Seqviewer) showing the gene annotation units (from chromosome 4, nucleotides 1,100,000 to 1,150,000). Each gene is label with an AGI locus ID. The lower part is the scores of this region by *PASS*. The double-headed arrows point to the relative location of the poly(A) sites and the peaks of *PASS *scores. The question marks indicate the regions of unknown gene annotation (see text for detail discussion on this). Note that the gene units on the reverse orientation are not predicted because *PASS *only predicts the sense direction as in mRNA.

One of the utilities of *PASS *is to predict poly(A) sites of unannotated genomic sequences, which could be helpful in genome annotation. This is because a poly(A) site marks the end of a 3'-UTR, which generally is the end of a gene. To test the effectiveness of *PASS *in this regard, we used it to scan several 50,000 nt genomic sequences downloaded from TAIR ([18];Arabidopsis Genome Initiative release 5). Figure 5 shows one of these examples, in which many poly(A) sites were predicted. When the gene annotation data (gene units, from TAIR) were overlaid with the *PASS *prediction scores, several interesting phenomena became obvious. The ends of the 6 genes annotated in this region (from left to right orientation only, since *PASS *scans one direction from 5' to 3' of the sequence) all have the relative high score at the 3' termini of their transcripts, particularly when comparing the scores in the coding region and 3'-UTR (e.g. AT4G02510 and AT4G02540, Figure 5). Some of them show a few sites with good scores in the 3'UTR (AT4g02500) or even in the coding sequences (AT4G02750), which may reflect alternative poly(A) sites. More interestingly, however, the two regions with the highest scores (marked with "?") were not located in any annotated genes. This could be due to the traditional annotation process failing to recognize the genes. Alternatively, there may be some special sequences that possess the features of a poly(A) site. It is also possible that *PASS *produces false positive sites. These possibilities could be distinguished using wet lab experiments (RT-PCR approach with oligo-dT and a sequence specific primer to detect transcript with a poly(A) tail, for example).

## Discussion

Based on the current model of Arabidopsis poly(A) signals and their features, we developed a GHMM-based algorithm that for the first time can predict poly(A) sites in plant mRNA. In this paper, the structure of the model is described, and the program was tested with known poly(A) sites. Using this model, we achieved sufficient sensitivity and specificity both at 97% in the coding sequence and random datasets at a threshold of 4. For other control datasets like 5' UTRs and introns, which are known to share some features with 3'UTRs, the *Sn *and *Sp *are still in a range of 72–82% at thresholds between 5.2 and 6. Moreover, the algorithm was able to predict many poly(A) site regions accurately when scanning a big fragment of Arabidopsis genomic sequences.

GHMM is an important model in gene identification and is widely used by gene identification software such as GENSCAN, GeneMarkS and HMMgene [19-21]. GHMM can give each state multiple observed values (instead of a single value to each state in HMM) which makes it more suitable for describing a model of biological sequences. This improvement, however, is at the expense of an increase in computation. For example, the calculation complexity of the Viterbi algorithm, a traditional HMM algorithm, is *O(N*^2^*L)*, in which N is the number of states and L is the length of a sequence, while the calculation complexity of GHMM is *O(N*^2^*L*^3^*/2)*. Such an improvement resulted in better sensitivity.

Graber et al. [5] described a HMM model that predicts the poly(A) sites in yeast. While the basic principles of HMM are used in modeling algorithms in which the parameters were designed rather than trained, the difference and improvement using GHMM can be found in our algorithm. Each of the two models deals with a distinct group of organisms both of which have different types of poly(A) signal conservation, from which different parameters have to be given. Our model was produced based on information of plant poly(A) signals from a much large dataset (from 8K, but also applicable to a dataset of 16,000 sequences, [6]). Moreover, the generalized HMM was used in our algorithm. GHMM is known for better detaching the main model from sub-modeling of each signal state, a function that is expandable for modeling complicated signals. Detailed comparisons of the differences between HMM and GHMM that was used in our algorithm can be found in additional files [see Additional file 1].

Liu et al. [22] used a machine learning method to generate human poly(A) signals and then used a support vector machine to identify the real sites. After refinement of their method, the sensitivity of their program increased from 56.3% to 94.4%, while its specificity reached 92.2%. Our results reached a similar level (although not by direct comparison), even though plant poly(A) signals are less conserved than those of humans. In particular, there are only 10 patterns that cover about 90% of NUE equivalent signals (53% being AAUAAA) in animals [23]. By contrast, in Arabidopsis, a list of such patterns reaches several hundreds, with no predominant patterns ([6]; Y-J. Shen and Q.Q. Li, unpublished observation). The prevalent signal AAUAAA, although it is the best, can only be found in about 10% of the plant genes [6]. The rest of hundreds of signal patterns form a continuous distribution without a clear cut-off value (Y-J. Shen and Q.Q. Li, unpublished observation). Even so, NUE is still the strongest signal among the tri-part poly(A) signals, including FUE and CE, based on classical genetics analysis [2].

Most recently, Cheng et al. [24] also reported a human poly(A) site prediction algorithm using a support vector machine. The algorithm took advantage of 15 highly conserved poly(A) signals, but also used other signals and U-rich elements to contribute to the prediction efficiency. These additional features improved the program's sensitivity, although the specificity remained more or less the same. Integrating new features like secondary structure into PASS should also improve its performance.

Beyond the known variability of the NUE signals in plants, a lack of conservation and identifiable features of other signal regions presents another difficulty in the prediction of poly(A) site by an algorithm. No highly conserved pattern was found in the FUE region. However, deletions of the FUE region were found to affect adjacent poly(A) site efficiency [2]. The best feature in the FUE that helped our program was the distinct T and A richness of the region [6]. The CE region also suffered a lack of sequence conservation. However, this region exhibited complex nucleotide profiles (See Additional file 2) that made feature selection easier. Under such circumstances, our program can predict many of the alterations of the poly(A) site efficiency in mutants constructed by conventional genetic means (Figure 4). In particular, upon the change of a few nucleotides within polyadenylation signals, *PASS *predicted the change of the poly(A) site usage efficiencies (Figure 4C and 4D) implying that the program has high merit in terms of accuracy.

*PASS *should also be useful in gene annotation, where DNA sequences can be entered and the poly(A) site profiles deduced. The high values of the *PASS *predictions are indicative of potential poly(A) sites which signify the end of a mature transcript. We demonstrated this possibility by scanning fragments of genomic DNA in 50,000 nts in length (though it can process longer sequences essentially without an upper limit) as shown in Figure 5. Furthermore, *PASS *can also be used to predict alternative poly(A) sites that are not normally found by EST experiments. Alternative polyadenylation has been found to be more frequent than what was originally anticipated in human (50%) and plant (25%) genes [23,25]. A complete understanding of the significance of alternative polyadenylation is yet to be realized. Our program should also be a useful addition towards achieving this goal.

## Conclusion

Based on the profiles of Arabidopsis polyadenylation signals, a new algorithm, named *PASS*, was developed to predict the poly(A) sites in plants. The efficacy of the program was tested using known poly(A) sites collected from EST sequencing projects or published papers. Interestingly, *PASS *can also predict the alterations of poly(A) site efficiency by traditional genetic mutations of poly(A) signals. Both specificity and sensitivity of the program reached around 97% at the best datasets. This algorithm will be useful in genome annotation by predicting the ends of the transcripts, in the study of alternative polyadenylation of mRNA, and in genetic engineering by enabling researchers to recognize and then eliminate potential undesirable poly(A) sites in the transgenes. The *PASS *program is available through our web site [26].

## Methods

### The datasets

The experimental dataset (also called the 8K dataset) used here has been described previously [6], and contains 8160 sequences from the genome of *Arabidopsis thaliana*. Briefly, all available expressed sequence tags (ESTs) were downloaded from GenBank, and those containing terminal poly(A) sequences [8 to 15 nucleotide (nt) with at least 80% adenine content] were recognized and trimmed. The terminal nt of each trimmed polyadenylated transcript was classified as the last nt before a poly(A) site. A total of 8160 such poly(A) sites were identified and confirmed through the comparison of genomic and EST sequences (The oligo(A) should not be found in the genomic sequence because these were added post-transcriptionally during the polyadenylation process). Using the poly(A) sites as a reference, the corresponding 400 nt genomic sequences were extracted in such a way that each sequence contained 301 nt upstream and 99 nt downstream of the poly(A) site. Thus, the poly(A) site in each sequence was between the 301^st ^nt and the 302^nd ^nt (from left to right; the poly(A) site was also the cleavage site. The cleavage reaction occurs between two nucleotides linked by a phosphodiester bond). The cleavage site is defined as the "0" position (note there is no nucleotide assigned to this position). Hence, the nucleotide sequences on the left (upstream) have a negative designation, and on the right have a positive (often omitted) designation. In general, for the purpose of easier description, the nucleotide on the left of the cleavage site (position 301 in the dataset) is normally referred to as the a poly(A) site.

This dataset was used to extract the features of the poly(A) signals and poly(A) sites. Other test sequences shown in the results were either downloaded from GenBank or from published papers as cited. For the *Sp *calculation, control datasets of the Arabidopsis coding (which do not include 5' and 3' UTRs and introns), 5'-UTR, and intron sequences were downloaded from The Arabidopsis Information Resources website (TAIR; [18]; Arabidopsis Genome Initiative Release 5, 2004). These sequences were trimmed into 400 nt in length each for better comparison with the 8K dataset. The coding sequence datasets were extracted from downloaded sequences in the range of 300–700 to avoid the inclusion of UTRs. The random sequences for the *Sp *calculation were generated based on the second order trinucleotide distribution [5] in the 8K dataset. For the genomic sequence scan, the Arabidopsis chromosome 4 genomic sequence was used (ATH1_chr4.1con.01222004; from TAIR). It is worth mentioning that the sequences used in this work are in DNA form, so nucleotides in these sequences are ATCG instead of AUCG as in RNA. This does not impact the analysis.

### Modeling routine

**Figure 6:**
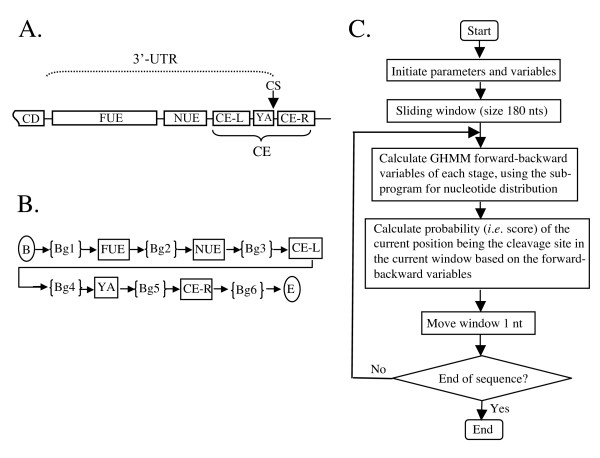
The structure of plant mRNA polyadenylation signals, the order of the GHMM, and a flowchart of *PASS*. A. A working model based on [6]. B. The order of GHMM. The arrowheads indicate the probability of changing of states (all probabilities were set to be 1). The rectangles represent regions with fixed length while the braces indicate regions with variable length. 3'-UTR, 3' untranslated region; CD, coding region; FUE, Far Upstream Element; NUE, Near Upstream Element; CE, cleavage element; CE-L, CE-R, Cleavage element left or right to the poly(A) site; CS, cleavage site, also known as poly(A) site; YA, represents TA or CA – predominant dinucleotides right before CS; B, beginning of the scan; Bg, background sequences between *cis*-elements; E, end of scan. Note that because YA is not found in all sequences, other dinucleotide combinations are also considered in GHMM. C. Flow chart of the *PASS *algorithm.

The topological structure is one of the most important factors in designing a GHMM model. The regular expression of topological structures in GHMM models is based on all connection structures, in which every state can go to any other state. This kind of topological structure does not take advantage of the positions of the signal elements in the 3' UTR (Figure 6A). Therefore, we employed a GHMM model that recognized the signals from left to right, and only allowed the recognition of signals from the current state to the next state in one direction, as indicated in Figure 6B. Based on the analysis of the current model of plant poly(A) signals [6], we classified the sequences into five regions (Figure 6A). The poly(A) signals are distributed in these regions with some spacing between the two signal elements. Based on this, we added a background state between the two signal states. To simplify the model, we assumed that the length of every signal was fixed but the length of background was variable. It was also possible that two signals were next to each other and thus the length of the background may be 0. The final model was designed in such a way that all calculations began on the first state and ended at the last state (Figure 6B). The order of the algorithm is shown in Figure 6C.

### Parameter setting

In this model, some basic parameters were set as follows: the number of states was 11 (Figure 6B, from Bg1 through Bg6); the array of signals in every state is set to be {A, T, C, G}. States in odd numbers were the background state with variable length, and states in even numbers were signal states with fixed length. Because the model begins with the first state and ends at the last state, the initial state distribution is set in π = {1,0,...,0}. Because every state (i) can be only transferred to the i+1 state, only the value of a_i,i+1 _is 1 in the state transition probability matrix, and other elements of the distribution matrix are 0. Other parameters such as distribution of nucleotides and length of state will be described below.

### Length of the signal elements

For modeling purposes, we needed to assign each state a few parameters including the signal nucleotide composition, signal pattern length, etc. To simplify the model, we assumed that the size or nucleotide length of each signal (FUE, NUE, CE, respectively) was fixed. As a first step, we had to designate reasonable signal lengths for each of them. To this end, we designed the following method to extract this data from the SignalSleuth experiments as described in Loke et al [6]. The observed total count of 3 nt patterns was used as a basis to calculate the "expected count" of pattern sizes of 4 nt, and the observed total count of 4 nt patterns was used to calculate the "expected count" of 5 nt, and so on. For example, the expected count of 4 nt patterns should decrease by 25% of the actual total count of 3 nt patterns because of an increase in length by one of the four nucleotides. The difference between the predicted count of patterns (random chance) and the actual observed count is useful in measuring pattern length uniqueness. The measure of the deviation from the randomness of the patterns offers a clue as to the potential length of the signals, because the real signals should have greater deviation from randomness than that of non-signals. As indicated on the histogram (see Additional file 3), the greatest difference observed for FUE is at 8 nt, NUE at 6 nt, CE-L at 6 nt, and CE-R at 7 nt, respectively. Importantly, using this bioinformatics approach, the signal lengths of the NUE and FUE match the lengths of NUE and FUE defined by classical genetic analysis, in which it was found that the NUE signal length is 6 nt, and the FUE is about 8 nt [2,27]. Note that the signal length is different from the range of signal region where the signal can be found. The latter is for modeling purposes, and is larger because it describes a collective area where the signals are found in different genes.

### Output probability of signal state

After determining the length of the signals, we needed to study the output probability *B *of the nucleotides (A, T, C, and G) in every signal state. To this end, we analyzed the distribution of nucleotides in each region (FUE, NUE and CEs) with the formula below, using the frequency data that was generated by SignalSleuth as described by Loke et al [6] using the 8K dataset.

Dε=∑i=1N(εi×Wi)∑ε∈{A,T,C,G}(∑i=1N(εi×Wi))
 MathType@MTEF@5@5@+=feaafiart1ev1aaatCvAUfKttLearuWrP9MDH5MBPbIqV92AaeXatLxBI9gBaebbnrfifHhDYfgasaacH8akY=wiFfYdH8Gipec8Eeeu0xXdbba9frFj0=OqFfea0dXdd9vqai=hGuQ8kuc9pgc9s8qqaq=dirpe0xb9q8qiLsFr0=vr0=vr0dc8meaabaqaciaacaGaaeqabaqabeGadaaakeaacqWGebardaWgaaWcbaacciGae8xTdugabeaakiabg2da9maalaaabaWaaabCaeaadaqadaqaaiab=v7aLnaaBaaaleaacqWGPbqAaeqaaOGaey41aqRaem4vaC1aaSbaaSqaaiabdMgaPbqabaaakiaawIcacaGLPaaaaSqaaiabdMgaPjabg2da9iabigdaXaqaaiabd6eaobqdcqGHris5aaGcbaWaaabuaeaacqGGOaakdaaeWbqaamaabmaabaGae8xTdu2aaSbaaSqaaiabdMgaPbqabaGccqGHxdaTcqWGxbWvdaWgaaWcbaGaemyAaKgabeaaaOGaayjkaiaawMcaaiabcMcaPaWcbaGaemyAaKMaeyypa0JaeGymaedabaGaemOta4eaniabggHiLdaaleaacqWF1oqzcqGHiiIZcqGG7bWEcqWGbbqqcqGGSaalcqWGubavcqGGSaalcqWGdbWqcqGGSaalcqWGhbWrcqGG9bqFaeqaniabggHiLdaaaaaa@6264@

where Wi=Ci∑s=1NCs
 MathType@MTEF@5@5@+=feaafiart1ev1aaatCvAUfKttLearuWrP9MDH5MBPbIqV92AaeXatLxBI9gBaebbnrfifHhDYfgasaacH8akY=wiFfYdH8Gipec8Eeeu0xXdbba9frFj0=OqFfea0dXdd9vqai=hGuQ8kuc9pgc9s8qqaq=dirpe0xb9q8qiLsFr0=vr0=vr0dc8meaabaqaciaacaGaaeqabaqabeGadaaakeaacqWGxbWvdaWgaaWcbaGaemyAaKgabeaakiabg2da9maalaaabaGaem4qam0aaSbaaSqaaiabbMgaPbqabaaakeaadaaeWbqaaiabdoeadnaaBaaaleaacqqGZbWCaeqaaaqaaiabdohaZjabg2da9iabbgdaXaqaaiabb6eaobqdcqGHris5aaaaaaa@3C88@ is the statistical weight of sequence *i*, and the more repeats this sequence has, the higher the weight is; *C*_*i *_is the frequency at which the *i*^th ^sequence occurs in this signal area; *D*_*ε *_represents the probability of the nucleotide *ε *in the signal element, *i.e*. the distribution of nucleotides, which is *ε *∈ {A, T, C, G}; *ε*_*i *_is the frequency of *ε *in the *i*^th ^sequence, where 1 ≤ *i *≤ *N*, and *N *is the number of signal patterns considered.

Taking signals in the FUE as an example, the representative nucleotide output probability *B *was calculated based on the top 50 patterns [see Additional file 4]. First, we calculated the weight of every pattern by count, and then calculated the repeat times *ε*_*i *_of every nucleotide in these patterns. The nucleotide output probability *B *for FUE hence are 0.0485, 0.7740, 0.0479 and 0.1290 for A, T, C, and G, respectively.

Using the same method, the nucleotide output probability *B *for CE-L and CE-R are: CE-L: 0.09987, 0.74970, 0.06186, 0.08860; CE-R: 0.08520, 0.78700, 0.07050, 0.05680 for A, T, C, and G, respectively.

The NUE signals are slightly better conserved than other signals, and the transition from one nt to the next may be constrained. To present these interactions of hexamer signals, we used a subset of first order inhomogeneous Markov model to describe the feature information. A frequency transport matrix was used to analyze the 50 most predominant NUE signals [6]. The equation is shown below:

PN=[PN(A/A)PN(T/A)PN(C/A)PN(G/A)PN(A/T)PN(T/T)PN(C/T)PN(G/T)PN(A/C)PN(T/C)PN(C/C)PN(G/C)PN(A/G)PN(T/G)PN(C/G)PN(G/G)]=[S(AA)∑AS(AT)∑AS(AC)∑AS(AG)∑AS(TA)∑TS(TT)∑TS(TC)∑TS(TG)∑TS(CA)∑CS(CT)∑CS(CC)∑CS(CG)∑CS(GA)∑GS(GT)∑GS(GC)∑GS(GG)∑G]
 MathType@MTEF@5@5@+=feaafiart1ev1aaatCvAUfKttLearuWrP9MDH5MBPbIqV92AaeXatLxBI9gBaebbnrfifHhDYfgasaacH8akY=wiFfYdH8Gipec8Eeeu0xXdbba9frFj0=OqFfea0dXdd9vqai=hGuQ8kuc9pgc9s8qqaq=dirpe0xb9q8qiLsFr0=vr0=vr0dc8meaabaqaciaacaGaaeqabaqabeGadaaakeaacqWGqbaucqWGobGtcqGH9aqpdaWadaqaauaabeqaeqaaaaaabaGaemiuaaLaemOta4KaeiikaGIaemyqaeKaei4la8IaemyqaeKaeiykaKcabaGaemiuaaLaemOta4KaeiikaGIaemivaqLaei4la8IaemyqaeKaeiykaKcabaGaemiuaaLaemOta4KaeiikaGIaem4qamKaei4la8IaemyqaeKaeiykaKcabaGaemiuaaLaemOta4KaeiikaGIaem4raCKaei4la8IaemyqaeKaeiykaKcabaGaemiuaaLaemOta4KaeiikaGIaemyqaeKaei4la8IaemivaqLaeiykaKcabaGaemiuaaLaemOta4KaeiikaGIaemivaqLaei4la8IaemivaqLaeiykaKcabaGaemiuaaLaemOta4KaeiikaGIaem4qamKaei4la8IaemivaqLaeiykaKcabaGaemiuaaLaemOta4KaeiikaGIaem4raCKaei4la8IaemivaqLaeiykaKcabaGaemiuaaLaemOta4KaeiikaGIaemyqaeKaei4la8Iaem4qamKaeiykaKcabaGaemiuaaLaemOta4KaeiikaGIaemivaqLaei4la8Iaem4qamKaeiykaKcabaGaemiuaaLaemOta4KaeiikaGIaem4qamKaei4la8Iaem4qamKaeiykaKcabaGaemiuaaLaemOta4KaeiikaGIaem4raCKaei4la8Iaem4qamKaeiykaKcabaGaemiuaaLaemOta4KaeiikaGIaemyqaeKaei4la8Iaem4raCKaeiykaKcabaGaemiuaaLaemOta4KaeiikaGIaemivaqLaei4la8Iaem4raCKaeiykaKcabaGaemiuaaLaemOta4KaeiikaGIaem4qamKaei4la8Iaem4raCKaeiykaKcabaGaemiuaaLaemOta4KaeiikaGIaem4raCKaei4la8Iaem4raCKaeiykaKcaaaGaay5waiaaw2faaiabg2da9maadmaabaqbaeqabqabaaaaaeaadaWcaaqaaiabdofatjabcIcaOiabdgeabjabdgeabjabcMcaPaqaamaaqababaaaleaacqWGbbqqaeqaniabggHiLdaaaaGcbaWaaSaaaeaacqWGtbWucqGGOaakcqWGbbqqcqWGubavcqGGPaqkaeaadaaeqaqaaaWcbaGaemyqaeeabeqdcqGHris5aaaaaOqaamaalaaabaGaem4uamLaeiikaGIaemyqaeKaem4qamKaeiykaKcabaWaaabeaeaaaSqaaiabdgeabbqab0GaeyyeIuoaaaaakeaadaWcaaqaaiabdofatjabcIcaOiabdgeabjabdEeahjabcMcaPaqaamaaqababaaaleaacqWGbbqqaeqaniabggHiLdaaaaGcbaWaaSaaaeaacqWGtbWucqGGOaakcqWGubavcqWGbbqqcqGGPaqkaeaadaaeqaqaaaWcbaGaemivaqfabeqdcqGHris5aaaaaOqaamaalaaabaGaem4uamLaeiikaGIaemivaqLaemivaqLaeiykaKcabaWaaabeaeaaaSqaaiabdsfaubqab0GaeyyeIuoaaaaakeaadaWcaaqaaiabdofatjabcIcaOiabdsfaujabdoeadjabcMcaPaqaamaaqababaaaleaacqWGubavaeqaniabggHiLdaaaaGcbaWaaSaaaeaacqWGtbWucqGGOaakcqWGubavcqWGhbWrcqGGPaqkaeaadaaeqaqaaaWcbaGaemivaqfabeqdcqGHris5aaaaaOqaamaalaaabaGaem4uamLaeiikaGIaem4qamKaemyqaeKaeiykaKcabaWaaabeaeaaaSqaaiabdoeadbqab0GaeyyeIuoaaaaakeaadaWcaaqaaiabdofatjabcIcaOiabdoeadjabdsfaujabcMcaPaqaamaaqababaaaleaacqWGdbWqaeqaniabggHiLdaaaaGcbaWaaSaaaeaacqWGtbWucqGGOaakcqWGdbWqcqWGdbWqcqGGPaqkaeaadaaeqaqaaaWcbaGaem4qameabeqdcqGHris5aaaaaOqaamaalaaabaGaem4uamLaeiikaGIaem4qamKaem4raCKaeiykaKcabaWaaabeaeaaaSqaaiabdoeadbqab0GaeyyeIuoaaaaakeaadaWcaaqaaiabdofatjabcIcaOiabdEeahjabdgeabjabcMcaPaqaamaaqababaaaleaacqWGhbWraeqaniabggHiLdaaaaGcbaWaaSaaaeaacqWGtbWucqGGOaakcqWGhbWrcqWGubavcqGGPaqkaeaadaaeqaqaaaWcbaGaem4raCeabeqdcqGHris5aaaaaOqaamaalaaabaGaem4uamLaeiikaGIaem4raCKaem4qamKaeiykaKcabaWaaabeaeaaaSqaaiabdEeahbqab0GaeyyeIuoaaaaakeaadaWcaaqaaiabdofatjabcIcaOiabdEeahjabdEeahjabcMcaPaqaamaaqababaaaleaacqWGhbWraeqaniabggHiLdaaaaaaaOGaay5waiaaw2faaaaa@2912@

where *PN*(*T*/*A*) is the probability of a transition from state "*A*" to "*T*"; *S*(*AT*) is the sum of times of this transport; Σ_*A *_= *S*(*AA*) + *S*(*AT*) + *S*(*AC*) + *S*(*AG*). The same rule was used for the others. Thus, we obtained parameters of the NUE sub-model as: probability distribution of the first nucleotide, *PN*_0 _= [0.6276, 0.3563, 0.0001, 0.0161]. The distributions of the second to sixth nucleotides are listed below, respectively,

PN1=[0.6215,0.3785,0.0001,0.00010.6788,0.3212,0.0001,0.00011,0.0001,0.0001,0.00011,0.0001,0.0001,0.0001],PN2=[0.5229,0.4464,0,0.03070.6003,0.2637,0.0441,0.09201,0.0001,0.0001,0.00011,0.0001,0.0001,0.0001],PN3=[0.5470,0.4258,0,0.02720.6700,0.2377,0.0418,0.05051,0.0001,0.0001,0.00011,0.0001,0.0001,0.0001]PN4=[0.6181,0.3584,0.0001,0.02350.7537,0.2463,0.0001,0.00011,0.0001,0.0001,0.00011,0.0001,0.0001,0.0001],PN5=[0.5550,0.4212,0.0001,0.02380.5277,0.4723,0.0001,0.00011,0.0001,0.0001,0.00011,0.0001,0.0001,0.0001]
 MathType@MTEF@5@5@+=feaafiart1ev1aaatCvAUfKttLearuWrP9MDH5MBPbIqV92AaeXatLxBI9gBaebbnrfifHhDYfgasaacH8akY=wiFfYdH8Gipec8Eeeu0xXdbba9frFj0=OqFfea0dXdd9vqai=hGuQ8kuc9pgc9s8qqaq=dirpe0xb9q8qiLsFr0=vr0=vr0dc8meaabaqaciaacaGaaeqabaqabeGadaaakeaafaqabeWacaaabaGaemiuaaLaemOta40aaSbaaSqaaiabigdaXaqabaGccqGH9aqpdaWadaqaauaabaqaeqaaaaaabaGaeGimaaJaeiOla4IaeGOnayJaeGOmaiJaeGymaeJaeGynauJaeiilaWcabaGaeGimaaJaeiOla4IaeG4mamJaeG4naCJaeGioaGJaeGynauJaeiilaWcabaGaeGimaaJaeiOla4IaeGimaaJaeGimaaJaeGimaaJaeGymaeJaeiilaWcabaGaeGimaaJaeiOla4IaeGimaaJaeGimaaJaeGimaaJaeGymaedabaGaeGimaaJaeiOla4IaeGOnayJaeG4naCJaeGioaGJaeGioaGJaeiilaWcabaGaeGimaaJaeiOla4IaeG4mamJaeGOmaiJaeGymaeJaeGOmaiJaeiilaWcabaGaeGimaaJaeiOla4IaeGimaaJaeGimaaJaeGimaaJaeGymaeJaeiilaWcabaGaeGimaaJaeiOla4IaeGimaaJaeGimaaJaeGimaaJaeGymaedabaGaeGymaeJamaiGamaaaiilaWcabaGaeGimaaJaeiOla4IaeGimaaJaeGimaaJaeGimaaJaeGymaeJaeiilaWcabaGaeGimaaJaeiOla4IaeGimaaJaeGimaaJaeGimaaJaeGymaeJaeiilaWcabaGaeGimaaJaeiOla4IaeGimaaJaeGimaaJaeGimaaJaeGymaedabiqaa83acqaIXaqmcWaGacTaaaGGSaalaeaacqaIWaamcqGGUaGlcqaIWaamcqaIWaamcqaIWaamcqaIXaqmcqGGSaalaeaacqaIWaamcqGGUaGlcqaIWaamcqaIWaamcqaIWaamcqaIXaqmcqGGSaalaeaacqaIWaamcqGGUaGlcqaIWaamcqaIWaamcqaIWaamcqaIXaqmaaaacaGLBbGaayzxaaGaeiilaWcabaaabaGaemiuaaLaemOta40aaSbaaSqaaiabikdaYaqabaGccqGH9aqpdaWadaqaauaabaqaeqaaaaaabaGaeGimaaJaeiOla4IaeGynauJaeGOmaiJaeGOmaiJaeGyoaKJaeiilaWcabaGaeGimaaJaeiOla4IaeGinaqJaeGinaqJaeGOnayJaeGinaqJaeiilaWcabaGaeGimaaJamaiG4kaaaiilaWcabaGaeGimaaJaeiOla4IaeGimaaJaeG4mamJaeGimaaJaeG4naCdabaGaeGimaaJaeiOla4IaeGOnayJaeGimaaJaeGimaaJaeG4mamJaeiilaWcabaGaeGimaaJaeiOla4IaeGOmaiJaeGOnayJaeG4mamJaeG4naCJaeiilaWcabaGaeGimaaJaeiOla4IaeGimaaJaeGinaqJaeGinaqJaeGymaeJaeiilaWcabaGaeGimaaJaeiOla4IaeGimaaJaeGyoaKJaeGOmaiJaeGimaadabaGaeGymaeJamaiGqlaaaiilaWcabaGaeGimaaJaeiOla4IaeGimaaJaeGimaaJaeGimaaJaeGymaeJaeiilaWcabaGaeGimaaJaeiOla4IaeGimaaJaeGimaaJaeGimaaJaeGymaeJaeiilaWcabaGaeGimaaJaeiOla4IaeGimaaJaeGimaaJaeGimaaJaeGymaedabaGaeGymaeJamaiGOlaaaiilaWcabaGaeGimaaJaeiOla4IaeGimaaJaeGimaaJaeGimaaJaeGymaeJaeiilaWcabaGaeGimaaJaeiOla4IaeGimaaJaeGimaaJaeGimaaJaeGymaeJaeiilaWcabaGaeGimaaJaeiOla4IaeGimaaJaeGimaaJaeGimaaJaeGymaedaaaGaay5waiaaw2faaiabcYcaSaqaaiabdcfaqjabd6eaonaaBaaaleaacqaIZaWmaeqaaOGaeyypa0ZaamWaaeaafaqaaeabeaaaaaqaaiabicdaWiabc6caUiabiwda1iabisda0iabiEda3iabicdaWiabcYcaSaqaaiabicdaWiabc6caUiabisda0iabikdaYiabiwda1iabiIda4iabcYcaSaqaaiabicdaWiadaci9caaacYcaSaqaaiabicdaWiabc6caUiabicdaWiabikdaYiabiEda3iabikdaYaqaaiabicdaWiabc6caUiabiAda2iabiEda3iabicdaWiabicdaWiabcYcaSaqaaiabicdaWiabc6caUiabikdaYiabiodaZiabiEda3iabiEda3iabcYcaSaqaaiabicdaWiabc6caUiabicdaWiabisda0iabigdaXiabiIda4iabcYcaSaqaaiabicdaWiabc6caUiabicdaWiabiwda1iabicdaWiabiwda1aqaaiabigdaXiadaciadaaacYcaSaqaaiabicdaWiabc6caUiabicdaWiabicdaWiabicdaWiabigdaXiabcYcaSaqaaiabicdaWiabc6caUiabicdaWiabicdaWiabicdaWiabigdaXiabcYcaSaqaaiabicdaWiabc6caUiabicdaWiabicdaWiabicdaWiabigdaXaqaaiabigdaXiadacigdaaacYcaSaqaaiabicdaWiabc6caUiabicdaWiabicdaWiabicdaWiabigdaXiabcYcaSaqaaiabicdaWiabc6caUiabicdaWiabicdaWiabicdaWiabigdaXiabcYcaSaqaaiabicdaWiabc6caUiabicdaWiabicdaWiabicdaWiabigdaXaaaaiaawUfacaGLDbaaaeaacqWGqbaucqWGobGtdaWgaaWcbaGaeGinaqdabeaakiabg2da9maadmaabaqbaeaabqabaaaaaeaacqaIWaamcqGGUaGlcqaI2aGncqaIXaqmcqaI4aaocqaIXaqmcqGGSaalaeaacqaIWaamcqGGUaGlcqaIZaWmcqaI1aqncqaI4aaocqaI0aancqGGSaalaeaacqaIWaamcqGGUaGlcqaIWaamcqaIWaamcqaIWaamcqaIXaqmcqGGSaalaeaacqaIWaamcqGGUaGlcqaIWaamcqaIYaGmcqaIZaWmcqaI1aqnaeaacqaIWaamcqGGUaGlcqaI3aWncqaI1aqncqaIZaWmcqaI3aWncqGGSaalaeaacqaIWaamcqGGUaGlcqaIYaGmcqaI0aancqaI2aGncqaIZaWmcqGGSaalaeaacqaIWaamcqGGUaGlcqaIWaamcqaIWaamcqaIWaamcqaIXaqmcqGGSaalaeaacqaIWaamcqGGUaGlcqaIWaamcqaIWaamcqaIWaamcqaIXaqmaeaacqaIXaqmcWaGacWaaaGGSaalaeaacqaIWaamcqGGUaGlcqaIWaamcqaIWaamcqaIWaamcqaIXaqmcqGGSaalaeaacqaIWaamcqGGUaGlcqaIWaamcqaIWaamcqaIWaamcqaIXaqmcqGGSaalaeaacqaIWaamcqGGUaGlcqaIWaamcqaIWaamcqaIWaamcqaIXaqmaeaacqaIXaqmcWaGaIXaaaGGSaalaeaacqaIWaamcqGGUaGlcqaIWaamcqaIWaamcqaIWaamcqaIXaqmcqGGSaalaeaacqaIWaamcqGGUaGlcqaIWaamcqaIWaamcqaIWaamcqaIXaqmcqGGSaalaeaacqaIWaamcqGGUaGlcqaIWaamcqaIWaamcqaIWaamcqaIXaqmaaaacaGLBbGaayzxaaGaeiilaWcabaGaemiuaaLaemOta40aaSbaaSqaaiabiwda1aqabaGccqGH9aqpdaWadaqaauaabaqaeqaaaaaabaGaeGimaaJaeiOla4IaeGynauJaeGynauJaeGynauJaeGimaaJaeiilaWcabaGaeGimaaJaeiOla4IaeGinaqJaeGOmaiJaeGymaeJaeGOmaiJaeiilaWcabaGaeGimaaJaeiOla4IaeGimaaJaeGimaaJaeGimaaJaeGymaeJaeiilaWcabaGaeGimaaJaeiOla4IaeGimaaJaeGOmaiJaeG4mamJaeGioaGdabaGaeGimaaJaeiOla4IaeGynauJaeGOmaiJaeG4naCJaeG4naCJaeiilaWcabaGaeGimaaJaeiOla4IaeGinaqJaeG4naCJaeGOmaiJaeG4mamJaeiilaWcabaGaeGimaaJaeiOla4IaeGimaaJaeGimaaJaeGimaaJaeGymaeJaeiilaWcabaGaeGimaaJaeiOla4IaeGimaaJaeGimaaJaeGimaaJaeGymaedabaGaeGymaeJamaiGymaaaiilaWcabaGaeGimaaJaeiOla4IaeGimaaJaeGimaaJaeGimaaJaeGymaeJaeiilaWcabaGaeGimaaJaeiOla4IaeGimaaJaeGimaaJaeGimaaJaeGymaeJaeiilaWcabaGaeGimaaJaeiOla4IaeGimaaJaeGimaaJaeGimaaJaeGymaedabaGaeGymaeJamaiGWmaaaiilaWcabaGaeGimaaJaeiOla4IaeGimaaJaeGimaaJaeGimaaJaeGymaeJaeiilaWcabaGaeGimaaJaeiOla4IaeGimaaJaeGimaaJaeGimaaJaeGymaeJaeiilaWcabaGaeGimaaJaeiOla4IaeGimaaJaeGimaaJaeGimaaJaeGymaedaaaGaay5waiaaw2faaaaaaaa@1DE0@

Therefore, for a certain hexamer sequence *S *= *s*_1_*s*_2_*s*_3_*s*_4_*s*_5_*s*_6 _we can calculate the probability of the NUE signal at the S position:

*P*[*S *| *NUE*] = *PN*_0_(*s*_1_)*PN_1_(*s*_2_|*s*_1_)*PN_2_(*s*_3_|*s*_2_)*PN_3_(*s*_4_|*s*_3_)*PN_4_(*s*_5_|*s*_4_)*PN_5_(*s*_6_|*s*_5_).

The same kind of first order inhomogeneous Markov model was established for the poly(A) site signal (YA in the model, Fig. 6A). For this, we randomly selected 1000 sequences from the 8K dataset and obtained the poly(A) site parameters listed blow. Initiated state *CSPN*_0 _= [0.0730,0.4630, 0.3250, 0.1390],

2^nd ^state CSPN1=[0.4247,0.3973,0.1096,0.06850.7171,0.1620,0.0648,0.05620.7785,0.1569,0.0431,0.02150.84890.09350.01440.0432]
 MathType@MTEF@5@5@+=feaafiart1ev1aaatCvAUfKttLearuWrP9MDH5MBPbIqV92AaeXatLxBI9gBaebbnrfifHhDYfgasaacH8akY=wiFfYdH8Gipec8Eeeu0xXdbba9frFj0=OqFfea0dXdd9vqai=hGuQ8kuc9pgc9s8qqaq=dirpe0xb9q8qiLsFr0=vr0=vr0dc8meaabaqaciaacaGaaeqabaqabeGadaaakeaacqaIYaGmdaahaaWcbeqaaiabb6gaUjabbsgaKbaakiabbccaGiabbohaZjabbsha0jabbggaHjabbsha0jabbwgaLjabbccaGiabdoeadjabdofatjabdcfaqjabd6eaonaaBaaaleaacqaIXaqmaeqaaOGaeyypa0ZaamWaaeaafaqabeabeaaaaaqaaiabicdaWiabc6caUiabisda0iabikdaYiabisda0iabiEda3iabcYcaSaqaaiabicdaWiabc6caUiabiodaZiabiMda5iabiEda3iabiodaZiabcYcaSaqaaiabicdaWiabc6caUiabigdaXiabicdaWiabiMda5iabiAda2iabcYcaSaqaaiabicdaWiabc6caUiabicdaWiabiAda2iabiIda4iabiwda1aqaaiabicdaWiabc6caUiabiEda3iabigdaXiabiEda3iabigdaXiabcYcaSaqaaiabicdaWiabc6caUiabigdaXiabiAda2iabikdaYiabicdaWiabcYcaSaqaaiabicdaWiabc6caUiabicdaWiabiAda2iabisda0iabiIda4iabcYcaSaqaaiabicdaWiabc6caUiabicdaWiabiwda1iabiAda2iabikdaYaqaaiabicdaWiabc6caUiabiEda3iabiEda3iabiIda4iabiwda1iabcYcaSaqaaiabicdaWiabc6caUiabigdaXiabiwda1iabiAda2iabiMda5iabcYcaSaqaaiabicdaWiabc6caUiabicdaWiabisda0iabiodaZiabigdaXiabcYcaSaqaaiabicdaWiabc6caUiabicdaWiabikdaYiabigdaXiabiwda1aqaaiabicdaWiabc6caUiabiIda4iabisda0iabiIda4iabiMda5aqaaiabicdaWiabc6caUiabicdaWiabiMda5iabiodaZiabiwda1aqaaiabicdaWiabc6caUiabicdaWiabigdaXiabisda0iabisda0aqaaiabicdaWiabc6caUiabicdaWiabisda0iabiodaZiabikdaYaaaaiaawUfacaGLDbaaaaa@A448@

Based on the same method, we calculated a dimer sequence *S *= *s*_1 _*s*_2 _and obtained the probability at S position: *P*[*S*|*CS*] = *CSPN*_0_(*s*_1_)* *CSPN*_1_(*s*_2_|*s*_1_).

### The background parameters

Apart from the signal regions, there is not much information on the background states. Therefore, the parameters for background states were relatively random. Based on this condition, we first analyzed several basic factors in the background and modified them accordingly. The nucleotide output probabilities of background states were calculated by counting the nucleotide distribution of the whole sequence. We tested the distribution of nucleotides in the region of -160 to +100 nt.

The most important factor in the background state is the length between two signal states. Taking the background states near the poly(A) site as an example, Bg4 and Bg5 are located upstream and downstream of the poly(A) site, and both the CE-L and CE-R signal states could be about 10 nt distance from the poly(A) site. Therefore, the length of the background state can be set to a range with 0 nt to 10 nt. The maximum length *D *was set to 10. However, because the length of the background could change slightly, we set all the lengths to a uniform distribution, which can be calculated by *P*_*i*_(*d*) = 1/(1+*D*_*i*_), where *P*_*i*_(*d*) is the probability of the length of the background *i*^th ^to be *d*, and *D*_*i *_is the possible maximum length of the *i*^th ^background. All background lengths were set by this method. The initial possible maximum length of Bg1, Bg2, Bg3, Bg4, Bg5 and Bg6 were set to 100, 100, 20, 10, 10 and 15, respectively.

To identify the background region, we needed to consider the near signal region of both sides. For example, Bg3 lies between NUE and CE-L signal states, the range of NUE state is 10~30 nt and CE-L signal region is from 1 to 10 nt. Therefore, the range of Bg3 could be set in the center of these two regions which is 6~20 nt. The distributions of the background region and nucleotide output probability *B *are listed in [Additional file 5].

### Formula for the output of scores

We applied a sliding 180 nt-wide window to calculate the output of scores for the sequences. For every nucleotide, our program computed a score in all windows that contained this nucleotide. The window slid along the entire sequence, combining values of forward-backward variables using the following equation for the output of the score at nucleotide *t*:

Score(t)=max⁡w{S(t)}S(t)={log⁡10PSw,t+120}/2
 MathType@MTEF@5@5@+=feaafiart1ev1aaatCvAUfKttLearuWrP9MDH5MBPbIqV92AaeXatLxBI9gBaebbnrfifHhDYfgasaacH8akY=wiFfYdH8Gipec8Eeeu0xXdbba9frFj0=OqFfea0dXdd9vqai=hGuQ8kuc9pgc9s8qqaq=dirpe0xb9q8qiLsFr0=vr0=vr0dc8meaabaqaciaacaGaaeqabaqabeGadaaakqaabeqaaiabdofatjabdogaJjabd+gaVjabdkhaYjabdwgaLjabcIcaOiabdsha0jabcMcaPiabg2da9maaxababaGagiyBa0MaeiyyaeMaeiiEaGhaleaacqWG3bWDaeqaaOWaaiWabeaacqWGtbWucqGGOaakcqWG0baDcqGGPaqkaiaawUhacaGL9baaaeaacqWGtbWucqGGOaakcqWG0baDcqGGPaqkcqGH9aqpdaGadeqaaiGbcYgaSjabc+gaVjabcEgaNnaaBaaaleaacqaIXaqmcqaIWaamaeqaaOGaemiuaaLaem4uam1aaSbaaSqaaiabdEha3jabcYcaSiabdsha0bqabaGccqGHRaWkcqaIXaqmcqaIYaGmcqaIWaamaiaawUhacaGL9baacqGGVaWlcqaIYaGmaaaa@5D81@

where *w *is all of the windows that include nucleotide *t*; *PS*_*w*,*t *_is the forward-backward algorithm's probability that nucleotide *t *is a poly(A) site in window *w*; the two constants, 120 and 2, are used to adjust the scores to be in a manageable range.

### Calculation of sensitivity and specificity

The formulas for *Sp *and *Sn *calculations are given in the Results. The methods for the compiling false positive and false negative numbers are shown here. We employed a user defined value called *threshold *in these calculations. At a given threshold value (*t*), the score at an nt must be at least *t *in order for that nt to be a predicted poly(A) site. The False Positive sites (*FP*) were calculated as following: for a sequence of interest, let *n *represent the total number of nucleotides; let *p *represent the number of true poly(A) sites with a score equal or larger than a given *t*; let *m *represent the number of all sites with a score equal or larger than *t*. Then, *FP *= *m-p*. As one can see, using the 8K dataset sequences to calculate *FP *requires that all poly(A) sites have to be identified. Due to the fact that the identification of true poly(A) sites in a given 3'UTR is incomplete in plants (many alternative poly(A) sites may not be represented in the EST collection, or the dataset is not sufficiently large enough), sequences that are known to not possess poly(A) sites were used to tally *FP*. These sequences include protein-coding sequences, 5'-UTRs, and introns as indicated in "The Datasets" under Methods. Random sequences generated by preserving the trinucleotide distribution were also used. In these control datasets, *FP *= *m*. For True Positive sites (*TP*), at a given *t*, in the sequences with known poly(A) sites, *TP *= *p*. In the control sequences, *TP *= *n-m*. False negative (*FN*) is the number of actual sites that cannot be identified or predicted correctly. To calculate *FN*, let *f *represent the number of true poly(A) sites with a score smaller than a given *t*. Hence, at a given *t*, in the sequences with known poly(A) sites, *FN *= *f*.

## Abbreviations

CaMV, cauliflower mosaic virus

CE, cleavage element

CS, cleavage site

*FN*, false negative

*FP*, false positive

FUE, far upstream element

GHMM, Generalized Hidden Markov Model

HMM, Hidden Markov Model

mRNA, messenger RNA

nt, nucleotide(s)

NUE, near upstream element

*PASS*, Poly(A) Site Sleuth program

poly(A), polyadenine

pre-mRNA, precursor mRNA

*rbcS*, rubisco small subunit gene

*Sn*, sensitivity

*Sp*, specificity

TAIR, the Arabidopsis Information Resources [18]

*TP*, true positive

UTR, untranslated region(s)

## Authors' contributions

GJ and QQL were responsible for the strategy, coordination, implementation of the project and manuscript preparation. JZ, RJ and YL implemented the algorithm, development of the code, and initial testing. XW modified the final code made it more efficient and helped revising the manuscript. YS and KMD tested the program with different genes and generated the output figures. JCL generated the signal length data. GJR implemented the algorithm in C++ which was used for testing, and participated manuscript writing.

## Supplementary Material

Additional File 1 Forward-backward Algorithm of GHMM Used in *PASS*. Details on how GHMM was implemented in the algorithm, including parameter settings and mathematical formulas. Click here for file

Additional File 2 The distribution of nucleotides in the 20 nt region around poly(A) sites. Fraction of each of the four nucleotides around poly(A) sites. Click here for file

Additional File 3 Analysis of the lengths of signal elements in FUE, NUE, CE-L and CE-R. Data showing the reason why the length of nucleotide sequences of each signal element was chosen. Click here for file

Additional File 4 Frequency of eight nucleotides patterns with high counts in FUE. Ranked list of the counts of the top 50 patterns found in FUE. Click here for file

Additional File 5 Statistical features of the background. Numeric data for setting background parameter. Click here for file
