# Supplementary material for: Predictive modeling of plant messenger RNA polyadenylation sites
Source: BMC Bioinformatics. 2007 Feb 7;8:43. doi: 10.1186/1471-2105-8-43 (PMC1805453; doi:10.1186/1471-2105-8-43)
Supplement: Additional File 1 — Forward-backward Algorithm of GHMM Used in PASS. Details on how GHMM was implemented in the algorithm, including parameter settings and mathematical formulas. [file 1471-2105-8-43-S1.pdf]

Forward-backward Algorithm of GHMM Used in *PASS*

● **Definition of parameters**

1) N： number of states in the model
2) M： number of symbols can be observed at each state
3) V={v_1,v_2,…, v_M}：set of observable discrete symbols
4) B={$b_j(k)$}：probability distribution of each observable symbols at state S_j
5) $b_j(k)$=P（v_K at t| $q_t = S_j$）, 1≤ j≤ N, 1≤k≤ M
6) π={π_i}：initial distribution of state

   π_i=P（$q_1 = S_i$）
7) T：length of observed sequence
8) S={S_1,S_2,….., S_N}：number of states in the model. Unlike HMM, GHMM uses q_r to express it's in the state of No. r.
9) A={a_{ij}}：the probability from state S_i transferring to S_j

   a_{ij}= P[$q_{r+1} = S_j \mid q_r = S_i$], 1≤ i, j≤N. and a_{ii}=0，1≤ i≤N
10) D={D_i}：the largest sustained time in the state S_i, 1≤ i≤N
11) P={$p_i(d)$}：the probability of the sustained time to be d in the state S_i, 1≤ i≤N，0≤ $d \le D_i$

● **Forward algorithm**

Based on the definition of GHMM, in an observed sequence O=$O_1\,O_2\ldots O_T$, suppose the first state starts from *t*=1; last state ends at *t*=*T*; and before the moment t there are *r* states: q_1, q_2, …, q_r. The sustained length of each state are d_1, d_2, …, d_r, and $\sum_{s=1}^{r} d_s$=*t*. Then define the forward variable $\alpha_t(i)$ as below:

$$\alpha_t(i) = P\,(O_1\,O_2\ldots O_t,\ S_i \text{ ends at } t \mid \lambda)$$

In a specific model λ, $\alpha_t(i)$ means the probability of having observed sequence $O_1\, O_2 \ldots O_t$ at the moment t when state i is ended.

Detailed algorithm is described as below:

Since the model can only start from the first state, the initiation formula is :

$$\alpha_t(1) = \pi_1\, p_1(t) \prod_{s=1}^{t} b_1(O_s) \qquad 1 \le t \le T$$

$$\alpha_1(i) = 0 \qquad\qquad 2 \le i \le N$$

The background states have the odd state number. Since length of the signal state cannot be 0, so we only consider the probability that transferred from previous state. The modified algorithm is:

$$\alpha_t(j) = \sum_{d=1}^{\min(D_j, t-1)} [\alpha_{t-d}(j-1) p_j(d) \prod_{s=t-d+1}^{t} b_j(O_s)]$$

$$2 \le t \le T,\ 2 \le j \le N \textit{ and } j \bmod 2 = 1$$

The signal states have the even state number. Since the length of signal state is fixed and that of the background state can be 0, we need to consider probability that transferred from previous background state and the one before it. The modified algorithm is:

$$\alpha_t(j) = \begin{cases} \alpha_{t-d}(j-1) p_j(d) \prod_{s=t-d+1}^{t} b_j(O_s) \\ \quad 2 \le t \le T,\ j = 2 \, with\_fixed\_d\_value \\ \alpha_{t-d}(j-2) p_{j-1}(0) p_j(d) \prod_{s=t-d+1}^{t} b_j(O_s) + \\ \alpha_{t-d}(j-1) p_j(d) \prod_{s=t-d+1}^{t} b_j(O_s) \\ 2 \le t \le T, 3 \le j \le N, j \bmod 2 = 0 \, with\_fixed\_d\_value \end{cases}$$

Moreover, since the model can only end at the last background state, GHMM model only consider the probability of observed value O at moment T in the last state N. The stopping algorithm is:

$$P(O|\lambda) = \alpha_T(N)$$

●**Backward algorithm**

Based on same reason, in backward algorithm, define backward variable $\beta_t(i)$ to P（$O_t \ldots O_T$ | S_i begins at t, λ），

In a specific model λ, $\beta_t(i)$ means the probability of having observed sequence $O_t \ldots O_T$ at the moment t when the model ends at state i.

The algorithm is listed below:

Since the model can only stop at the last state, the initiation formula is set to be:

$$\beta_t(N) = p_N(T-t+1)\prod_{s=t}^{T} b_N(O_s) \qquad 1 \le t \le T$$

$$\beta_T(i) = 0 \qquad 1 \le i \le N-1$$

The background states have the odd state number. Since signal state cannot be 0, so we only consider the probability that transferred from previous state. The modified algorithm is:

$$\beta_t(j) = \sum_{d=1}^{\min(D_j, T-t)} \left[\prod_{s=t}^{t+d-1} b_j(O_s)\, p_j(d)\, \beta_{t+d}(j+1)\right]$$

$1 \le t \le T-1$, $1 \le j \le N-1$ and $j \bmod 2 = 1$

The signal states have the even state number. Since the length of signal state is fixed and the background state can be 0, we need to consider probability that transferred from previous background state and the one before it. The modified algorithm is:

$$\beta_t(j) = \begin{cases} p_j(d)\prod_{s=t}^{t+d-1} b_j(O_s)\beta_{t+d}(j+1) \\ \quad 1 \le t \le T-1,\ j = N-1 with\_fixed\_dvalue \\ p_j(d)\prod_{s=t}^{t+d-1} b_j(O_s) p_{j+1}(0)\beta_{t+d}(j+2) + p_j(d)\prod_{s=t}^{t+d-1} b_j(O_s)\beta_{t+d}(j+1) \\ \quad 1 \le t \le T-1, 1 \le j \le N-2, j \bmod 2 = 0 and\_dvalue\_is\_fixed \end{cases}$$

Moreover, since the model can only start at the first background state, GHMM model only consider the probability of observed value O at the first state. The

stopping algorithm is:

$$P（O|\lambda）=\beta_1(1)$$

Therefore, given a sequence O and model λ, the formula to calculate the probability of state i ending at the position t of sequence O is: $P(S_i \text{ ends at t}, S_{i+1} \text{ begins at t+1}|O,\lambda) = \frac{\alpha_t(i)\beta_{t+1}(i+1)}{P(O|\lambda)}$. Based on our model, the probability of position t to be poly(A) site is $\frac{\alpha_t(8)\beta_{t+1}(9)}{P(O|\lambda)}$.
