# Supplementary material for: Predictive modeling of plant messenger RNA polyadenylation sites
Source: BMC Bioinformatics. 2007 Feb 7;8:43. doi: 10.1186/1471-2105-8-43 (PMC1805453; doi:10.1186/1471-2105-8-43)
Supplement: Additional File 2 — The distribution of nucleotides in the 20 nt region around poly(A) sites. Fraction of each of the four nucleotides around poly(A) sites. [file 1471-2105-8-43-S2.pdf]

Additional file 2

The distribution of nucleotides in the 20 nt region around poly(A) sites

Position	-10	-9	-8	-7	-6	-5	-4	-3	-2	-1
A	0.229	0.207	0.203	0.201	0.222	0.229	0.235	0.153	0.059	0.733
T	0.485	0.531	0.550	0.523	0.468	0.452	0.411	0.490	0.488	0.163
C	0.136	0.122	0.122	0.139	0.151	0.151	0.194	0.217	0.313	0.060
G	0.149	0.140	0.125	0.137	0.159	0.168	0.161	0.140	0.140	0.044
Position	1	2	3	4	5	6	7	8	9	10
A	0.469	0.306	0.280	0.286	0.251	0.263	0.247	0.228	0.216	0.225
T	0.342	0.434	0.410	0.394	0.413	0.406	0.410	0.431	0.433	0.429
C	0.108	0.138	0.159	0.163	0.173	0.159	0.155	0.153	0.165	0.180
G	0.081	0.122	0.151	0.157	0.163	0.171	0.188	0.188	0.186	0.166
