# Supplementary material for: Predictive modeling of plant messenger RNA polyadenylation sites
Source: BMC Bioinformatics. 2007 Feb 7;8:43. doi: 10.1186/1471-2105-8-43 (PMC1805453; doi:10.1186/1471-2105-8-43)

Additional file 3. Analysis of the lengths of signal elements in FUE, NUE, CE-L and CE-R. Vertical value represents the length of the elements and the horizontal value represents the percentage of the difference between the actual number and the expected number.

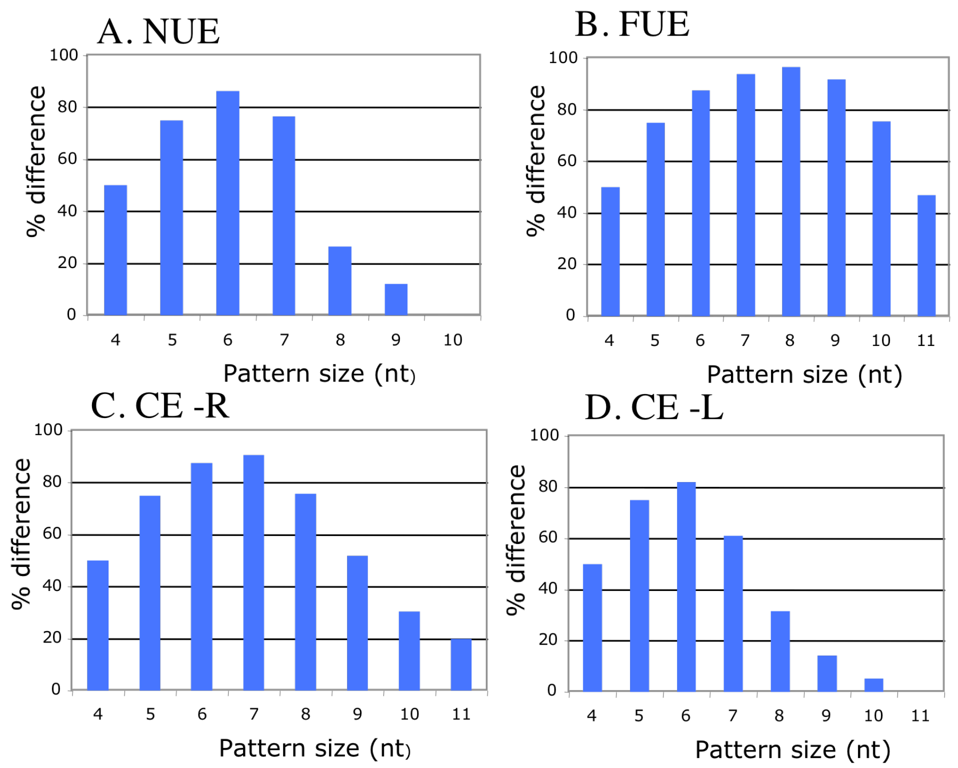

Supplement: Additional File 3 — Analysis of the lengths of signal elements in FUE, NUE, CE-L and CE-R. Data showing the reason why the length of nucleotide sequences of each signal element was chosen. [file 1471-2105-8-43-S3.pdf]
