# Supplementary material for: Predictive modeling of plant messenger RNA polyadenylation sites
Source: BMC Bioinformatics. 2007 Feb 7;8:43. doi: 10.1186/1471-2105-8-43 (PMC1805453; doi:10.1186/1471-2105-8-43)
Supplement: Additional File 4 — Frequency of eight nucleotides patterns with high counts in FUE. Ranked list of the counts of the top 50 patterns found in FUE. [file 1471-2105-8-43-S4.pdf]

Additional file 4:

Frequency of eight nucleotides patterns with high counts in FUE

NO 1-17		NO 18-34		NO 35-50	
Sequence	Count	Sequence	Count	Sequence	Count
tttgtttt	1024	ttttttc	598	tgtgtttt	475
ttttgttt	1018	ttttattt	577	gtttgttt	472
tttttttt	987	ttgttgtt	577	ttttggtt	464
tttttgtt	862	ttcttctt	568	ttttttta	460
tttttctt	806	gaagaaga	559	ttgtttgt	460
tttctttt	773	aagaagaa	557	tgtttctt	457
ttttcttt	759	tttatttt	536	tttgtgtt	449
ttgttttt	745	agaagaag	515	tgttgttt	440
ttcttttt	701	tttgttgt	513	tttgtttc	436
tttttttg	698	tttgtttg	502	ttttgtgt	434
tgtttttt	678	ttgttttg	500	tttgattt	434
tttggttt	666	tgttttgt	494	ttgtgttt	428
cttttttt	652	attttttt	491	tcttcttt	427
gttttttt	634	tgtttttg	489	tcttcttc	425
ttttttct	624	tttcttct	488	gtttttgt	424
ttttttgt	615	gttttgtt	487	cttttgtt	420
tctttttt	606	tgtttgtt	486		
