# Supplementary material for: Predictive modeling of plant messenger RNA polyadenylation sites
Source: BMC Bioinformatics. 2007 Feb 7;8:43. doi: 10.1186/1471-2105-8-43 (PMC1805453; doi:10.1186/1471-2105-8-43)
Supplement: Additional File 5 — Statistical features of the background. Numeric data for setting background parameter. [file 1471-2105-8-43-S5.pdf]

Additional file 5:

Statistical features of the background

	Region (nt)	A ratio	T ratio	C ratio	G ratio
Bg1	-160 ~ -81	0.272	0.381	0.159	0.188
Bg2	-80 ~ -21	0.296	0.401	0.139	0.165
Bg3	-6 ~ -20	0.324	0.401	0.126	0.149
Bg4	-1 ~ -5	0.179	0.462	0.205	0.154
Bg5	+1 ~ +5	0.414	0.348	0.126	0.111
Bg6	+10 ~ +20	0.276	0.407	0.164	0.153
